# Synthesis and Characterization of Recycled-TiC Reinforced AlZnMgCu Powder Metallurgy Composites

**DOI:** 10.3390/ma17194773

**Published:** 2024-09-28

**Authors:** Keerthivasan Navaneethakrishnan, Anandakrishnan Veeramani, Bharat Kumar Chigilipalli, Muralimohan Cheepu

**Affiliations:** 1 Department of Production Engineering, National Institute of Technology, Tiruchirappalli 620015, India; keerthinitt@gmail.com; 2 Department of Mechanical Engineering, Vignan’s Institute of Information Technology (A), Visakhapatnam 530049, India; chbharat334@gmail.com; 3 Department of Materials System Engineering, Pukyong National Engineering, Busan 46722, Republic of Korea; 4 Vitzronextech Co., Ltd., Ansan 425833, Republic of Korea

**Keywords:** AlZnMgCu matrix composite, corrosion behavior, mechanical properties, powder metallurgy, retrieved/recycled TiC, tribology

## Abstract

Recycling’s value in conserving scarce resources, avoiding environmental damage to the land, and reducing energy consumption is well known. This research aims to develop a composite that uses recycled reinforcement that was formed through an in situ method to build confidence in the usage of recycled materials. Thus, in connection with defense and aerospace industry applications, aluminum composite alloys receive more interest due to their light weight and high strength with improved mechanical properties; therefore, this research focuses on the fabrication of in situ-developed recycled TiC (r-TiC)-reinforced AlZnMgCu composites, i.e., new recycled materials. Experiments were conducted to determine the synthesized composites’ microstructural, mechanical, tribological, and corrosion properties. The microstructural study showed that r-TiC was distributed uniformly along the grain boundaries until the addition of 12% r-TiC. However, the accumulation of reinforcements began at 14% r-TiC addition and became more aggregated with subsequent increases in the percentage addition of r-TiC. The mechanical and tribological tests showed that the composite with 14% r-TiC was superior to all other compositions, with 60% improved mechanical qualities and the lowest wear rate of 0.0007 mm3/m. Composites containing 2% r-TiC showed the best corrosion resistance, an increase of 22% over AlZnMgCu, without reinforcement.

## 1. Introduction

Aluminum alloy has substituted most of the applications of steel, with its most significant advantage being its high strength-to-weight ratio [1]. AA7075 is one of the alloys in the AlZnMgCu classification that have the most utility in various fields, like aerospace, defense, sports equipment, etc. [2]. This zinc-based aluminum alloy has the most remarkable mechanical, physical, and chemical properties, often encouraging the researcher to further elevate the features. Titanium is the ninth available metal in the Earth’s crust, with its carbide TiC being crucial due to its high melting point, hardness, and resistance to wear/corrosion. Incorporating TiC particles into an aluminum matrix improves the material’s mechanical properties, including strength, rigidity, and wear resistance. Because of this, the demand for composite materials rather than naturally available materials is increasing daily [3,4,5]. During the synthesis of metal matrix composites with TiC addition, due to high cost and aggregation issues, instead of direct TiC addition, attempts are made to develop in situ TiC particle reinforcement in various matrix materials by adding halide salts [6,7,8]. However, in situ-developed reinforced composites leave more scraps in the laboratory, with the agglomeration made of reinforcement particles associated with the excessive addition of halide salts beyond a certain amount of percentage reinforcements due to various issues, which is unavoidable in certain circumstances. Recycling such wasted reinforcements from the composites conserves resources reduces waste and saves energy in high-performance applications. TiC recycling also facilitates more responsible resource management while lowering the price of developing new TiC-based products.

Hydrometallurgical TiC recycling uses chemical leaching [9] to recover TiC from TiC-based composite materials by dissolving them in acids or other chemical solvents. Combining this approach with others, such as pyrometallurgical and mechanical recycling [10], can improve reinforcement recovery and purity. The recovery and recycling process of TiC reinforcements from Ferro-Titanit MMC scraps using the hydrometallurgical technique were then reused to develop the Ferro-Titanit recycled MMCs [11,12]. Another method of reinforcement recovery called supergravity-induced separation was used to recover SiC particles from scrap aluminum matrix composites [13]. A chemical corrosion technique was used to extract TiC from the self-propagating combustion-synthesized Al-Ti-C system, and the extracted TiC’s morphology was studied [8]. Their study revealed that the grain size of the recycled reinforcement particles is not uniform after the recovery process. Variations in size for recycled reinforcements were also observed by Mourad et al. [14]. Alumina, SiC, and graphite were used to prepare hybrid reinforcements to create MMCs. However, their study revealed that recycled alumina and graphite exhibited better boning strength with matrix material. Tensile strength and wear resistance also noticeably increased with the addition of recovered reinforcement particles.

Among the various metal matrix composite-synthesizing techniques, powder metallurgy is well known for its major advantages, like precise control of reinforcement distribution, improved material properties, and the ability to produce complex shapes with high dimensional accuracy. Shanka et al. made an aluminum composite reinforced with silicon carbide through the powder metallurgy technique and reported that the reinforcements were distributed homogeneously and controlled through this technique [15]. Saravanan et al. improved the mechanical and wear properties of an AA7075 matrix using titanium carbide reinforcement addition through the powder metallurgy technique [16]. Satyanarayana et al. optimized the friction stir process parameters to enhance the joint strength of AA8011, which is reinforced with SiC nanoparticles [17]. Morumpudi et al. enhanced the corrosion resistance of an AA6061 matrix by adding ZrB_2_ reinforcement via the powder metallurgy route [18]. 

The recovered TiC particles were also characterized using standard metallurgical testing techniques. A higher amount of agglomerated TiC particles formed in the unusable scrap due to the excessive halide salt addition was recovered by super-induced gravity separation combined with a corrosive chemical extraction process [19]. Manohar et al. [20] and Maleki et al. [21] explained that the matrix particles and reinforcement particles facilitated the Orowan strengthening mechanism due to reinforcement and interfaces in their study. Golla et al. [22] prepared TiC-reinforced aluminum MMCs through the casting process and performed metallurgical and mechanical tests. The study involved the addition of TiC in 0, 2, 4, 6, and 8 wt.%, and it was found that the addition of 8% TiC in the aluminum matrix yielded a 32% increase in hardness and an 80% increase in yielding strength. Bharat et al. noticed that the secondary particles improved the wear resistance, but decreased the corrosive nature [23]. Nallusamy et al. [24] prepared AA7075/ZrB2 in situ composites and found noticeable increases in mechanical properties such as microhardness, yield strength, and tensile strength. Samuel M prepared recycled aluminum-alloy reinforcements from scrap with saffil ceramic fibers and aluminum alloys with a hot extrusion route [25]. The 10 vol.% Al_2_O_3_ saffil fibers produced a 77% increase in ultimate tensile strength (UTS) and an 86% increase in yield strength (YS) at 270 °C. The Vickers hardness and the elastic modulus were also found to increase at ambient temperature. This is because the resulting composites contained fibers that had very good internal cohesion. 

From the above literature, it is evident that the researchers are now focusing on green, highly efficient methods, and the low cost of recycling MMCs is increasing the demand and need for studying metallurgical and mechanical properties. Hence, in the present study, the focus lies on recovering TiC particles from scrap material produced during the stir-casting process, and the recovered TiC is again used as a reinforcement for producing aluminum composites using the powder metallurgy method. This study helps to investigate the r-TiC performance for the purpose of producing composite materials. Aerospace-grade aluminum alloy AlZnMgCu is used as a matrix material, and recycled TiC (denoted as r-TiC in this article) particles are used for reinforcement. MMCs are prepared using the powder metallurgy method. Recycled TiC (denoted as r-TiC in this article) particles are added as reinforcements in steps of two percentages to the AlZnMgCu matrix. The produced MMCs are subjected to metallurgical and mechanical tests such as microstructural analysis, porosity, compression, hardness, wear, and corrosion analysis. The results of the current research provide the basic results of recycled titanium carbide and AlZnMgCu composite for the selection of a material for suitable application in the aerospace and automobile industries, as per industrial needs.

## 2. Experimental Procedure

### 2.1. Recovery of TiC

**Figure 1:**
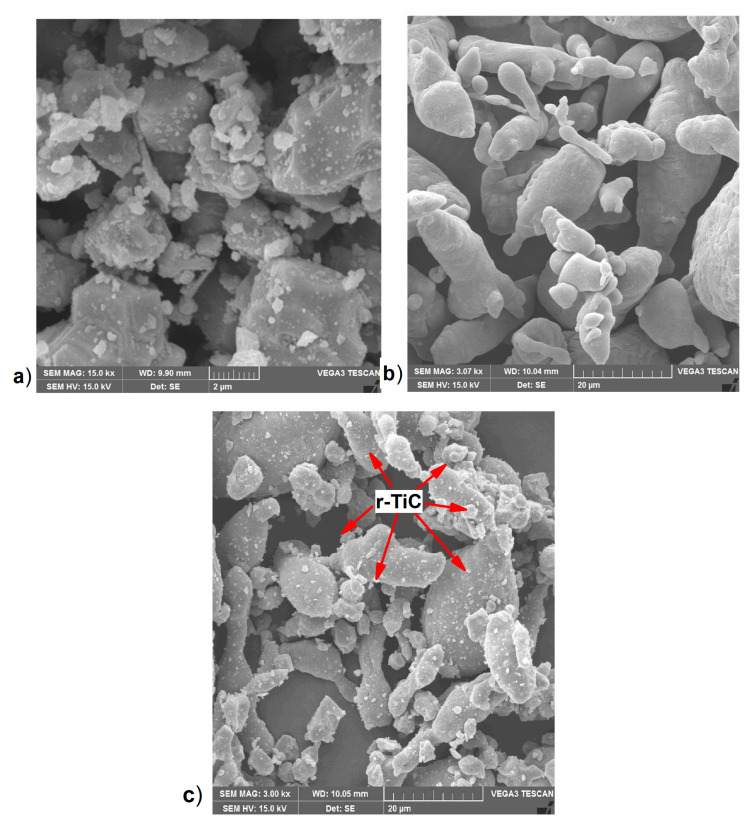
(**a**) SEM image of extracted r-TiC powder. (**b**) SEM image of AlZnMgCu alloy powder. (**c**) SEM image of AlZnMgCu + 14% r-TiC powder blend.

The unusable in situ stir-casted AA7075+TiC [26,27] composites obtained with high slag inclusion and called scrap AA7075+TiC were used to extract recycled titanium carbide (r-TiC) particles. The scrap was subjected to a super-induced gravity separation technique to separate 85% of the matrix, with the rest of the reinforcement becoming residue. The residue was further subjected to a corrosive chemical extraction process using 35–37% concentrated hydrochloric acid to extract r-TiC reinforcements [28]. A Vega3-Tescan scanning electron microscope from bruno, Czech Republic was used for the microstructural analysis of the extracted r-TiC as shown in Figure 1a. 

### 2.2. Blending, Compaction, and Sintering

To synthesize AlZnMgCu matrix r-TiC reinforcement composites using the powder metallurgy technique, AlZnMgCu alloy powder of mesh size 325 was bought from Thermal Fisher Scientific India Ltd., Mumbai, India. XRF analysis of the powder revealed 5.21% zinc, 2.41% magnesium, and 1.29% copper, which confirmed this as AA7075 alloy powder in Olympus InnovX2000 XRF analyser from Tokyo, Japan. The scanning electron microscopic image of this is shown in Figure 1b. Further, the AlZnMgCu matrix powder and the r-TiC reinforcement powders were blended in respective weight ratios to synthesize the AlZnMgCu + Y% (0, 2, 4, 6, 8, 10, 12, 14, and 16 wt. %) r-TiC composites. Based on laboratory trials and due to a lack of green strength, the r-TiC percentage was limited to 16% addition. 

Blending was carried out using an Insmart Systemsfrom hydrebad, India high-energy planetary ball mill without balls to retain the original as-received structure of the powder particles at 200 rpm for half an hour [29]. The blends had a uniform distribution of reinforcement over the matrix, as shown in Figure 1c.

**Figure 2:**
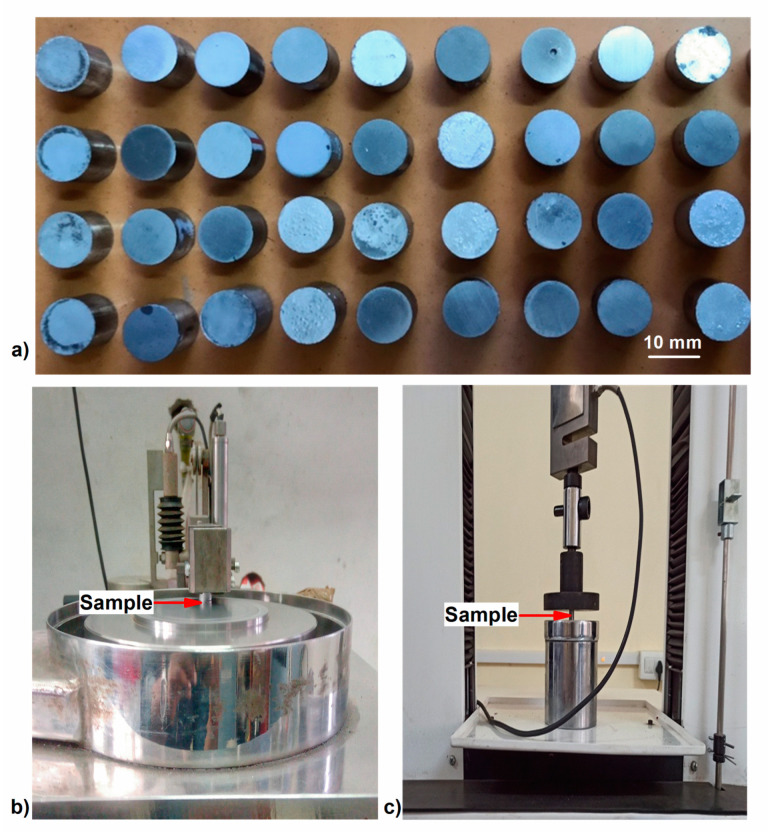
(**a**) A set of AlZnMgCu +Y% r-TiC composite samples. (**b**) Pin-on-disc dry sliding testing machine with AlZnMgCu +Y% r-TiC sample. (**c**) Universal testing machine with AlZnMgCu + Y% r-TiC sample.

Further, using a 100-ton universal testing machine (FIE-UTN100 from surat, India) along with a customized die, the blended composite powder mix was compressed at 450 MPa load to obtain cylindrical billets of 10 mm diameter and 10 mm height. Finally, the compacts were sintered using a muffle furnace at 600 °C for 3 h in an inert gas (argon) atmosphere to avoid oxidation, after which they were furnace-cooled to obtain AlZnMgCu +Y% r-TiC composite billets, as shown in Figure 2a.

### 2.3. Metallography and Density

The Rigaku-Ultima IV X-ray diffraction from Tokyo, Japan apparatus with specifications of a 0.02°/s scanning rate, 40 kV, 30 mA, 20 to 100° Bragg angles, and a Cu target of 1.5406 Å K_α_ radiation was used to analyze the presence of r-TiC, and to identify the intermetallic phases formed during sintering. Powder metallurgy samples were cut to the desired dimensions and installed using a METCO Mettio 1000 automatic hot mounting press (Chennai, India). The mounted samples were then ground and polished using a METCO Automet 250 from Chennai, India grinder–polisher machine. The samples were ground using abrasive papers of 400 to 2000 grit size and mechanically polished in three steps for 300 s using diamond suspension. In line with ASTM E3-01 [30], the finely polished samples were subjected to etching using Keller’s reagent. An Olympus optical microscope from Tokyo, Japan and a FESEM with EDS (Carl Zeiss, Gemini 300, Munich, Germany) were used to investigate the homogenous distribution and the presence of reinforcement r-TiC in the sintered composites. The density of the sintered compacts was measured using a density kit attached to an electronic weighing machine (CONTECH Precision CA Series, Pune, India) using Archimedes’ principle.

### 2.4. Hardness, Compression, and Wear Test

All composites synthesized had their microhardness measured at eight instances using a Vickers hardness tester (Wolpret Wilson 402HVD, Aachen, Germany) for 10 s with 0.5 kgf force. The average was used for the investigation. The average results were obtained. The wear test was carried out utilizing the pin-on-disk wear test setup (Model: TR20-LE, Ducom, Bangalore, India), as illustrated in Figure 2b, using the developed composite as a pin and the EN31 steel with 60HRC as a disk. The compression strength of the composite was also measured thrice for all composites at a strain rate of 1 mm/min using a Tech-SolM50 (Surat, India), a 50 kN universal testing machine, as illustrated in Figure 2c, according to E9-19 ASTM standards [31]. Variables of low load (9.81N)—LL, high load (29.43N)—HL, low sliding velocity (1 m/s)—LV, and high sliding velocity (3 m/s)—HV in all possible combinations were considered, keeping the sliding distance constant at 2000 m [26] to study the wear behavior at extreme conditions with minimal experiments. The mass loss method was used to compute the wear rate by measuring mass losses of the pin specimens to the nearest 0.1 mg after a wear run. The friction force between the pin (developed composite) and the disc (EN31 steel with 60HRC) was obtained using the Nanotech 2010 data acquisition system. It was then divided by the applied load to calculate the friction coefficient. To further discuss the wear analysis, elemental mapping of the composite was undertaken in an FESEM with EDS Carl Zeiss, Gemini 300, Munich, Germany. To explain both compression and wear, scanning electron images were obtained using the Vega3 Tescan instrument from Tokyo, Japan.

### 2.5. Corrosion Test

The synthesized AlZnMgCu + r-TiC composite’s electrochemical behavior was evaluated using a typical three-electrode cell PARC multi-channel potentiostat. After one hour of OCP (open circuit potential) stabilization at a potential range of −0.725 to 0.0 V and a scan rate of 0.5 mV/s, potentiodynamic polarization (PDP) curves were produced. Tafel extrapolation on the PDP curves in accordance with the G5-14 ASTM standard [32] resulted in Ecorr, the corrosion potential, and Icorr, the corrosion current density values for all nine composites. In addition, the corrosion rate was computed using the Icorr values as per the ASTM G102-89 standard [33].

## 3. Result and Discussion

### 3.1. Metallurgical Characterization of the Extracted r-TiC

The reinforcement r-TiC was successfully extracted from a scrap stir-casted in situ AA7075+TiC composite by means of super-induced gravity separation and corrosive chemical extraction processes, which were then subjected to XRD and XRF analyses. In the extraction process, 95 percent of the in situ-developed TiC and 85 percent of the matrix aluminum alloy were successfully recovered. The results revealed that the r-TiC peaks matched the TiC (JCPDS no. 98-010-3607) peaks, while the XRF results revealed 99% purity [28]. Further, it had an average particle size of 2 μm and an irregular shape, as shown in Figure 1a.

### 3.2. Characterization of the Composite

#### 3.2.1. Metallographic Analysis

**Figure 3:**
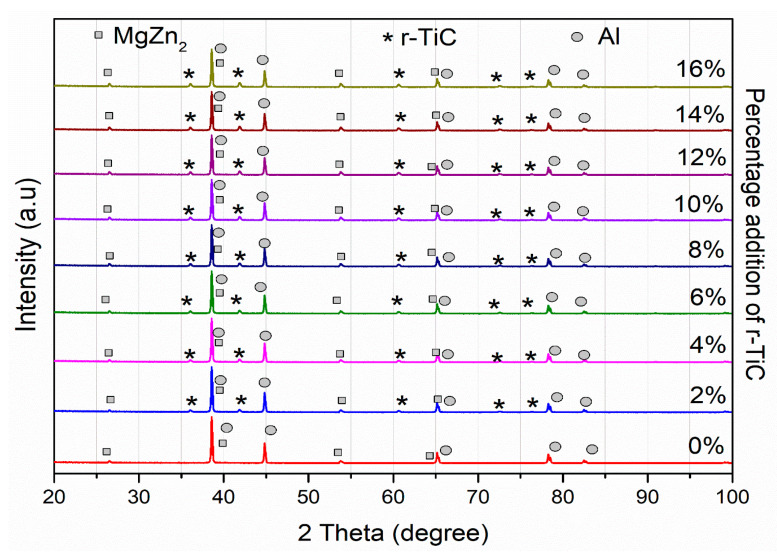
XRD of AlZnMgCu + Y% r-TiC composites.

The successfully synthesized AlZnMgCu +Y% r-TiC composites were subjected to X-ray diffraction analyses to identify the reinforcements and intermetallic phases of the nine samples, as shown in Figure 3. The highest-intensity peaks in all nine patterns were for the AlZnMgCu matrix and the recycled titanium carbide (r-TiC) reinforcement at 2θ–38.66° and 41.73°, respectively. We further alloyed elements in high concentrations, such as zinc and magnesium, and their matrix phases appeared as low-magnitude peaks. The matrix AlZnMgCu peaks revealed lattice planes like (111), (200), (220), (311), and (222) planes; similarly, lattice planes of titanium carbide were identified as (1 1 1), (0 0 2), (0 2 2), (1 1 3), and (2 2 2) from the peaks. Again, lattice planes for the intermetallic phases of MgZn2 were (1 1 2) and (0 2 1), as identified by the high-intensity peaks. The Malvern P analytical’s Xpert HighScore plus software (2009) identified the peaks and compared them with standard diffraction patterns (Al- 98-006-2688 and TiC-98-011-2564) in the Joint Committee on Powder Diffraction Standards (JCPDS) database. The peaks indicated that the atomic arrangement of the materials was a face-centered cubic.

**Figure 4:**
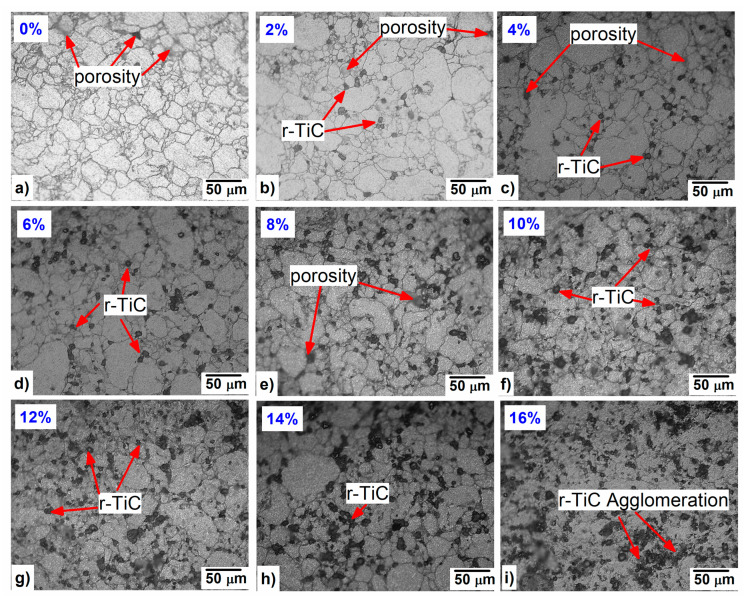
Optical micrograph of AlZnMgCu + Y% r-TiC (Y = 0, 2, 4, 6, 8, 10, 12, 14, 16). Composites (**a**) Y = 0%, (**b**) Y = 2%, (**c**) Y = 4%, (**d**) Y = 6%, © Y = 8%, (**f**) Y = 10%, (**g**) Y = 12%, (**h**) Y = 14%, (**i**) Y = 16%.

**Figure 5:**
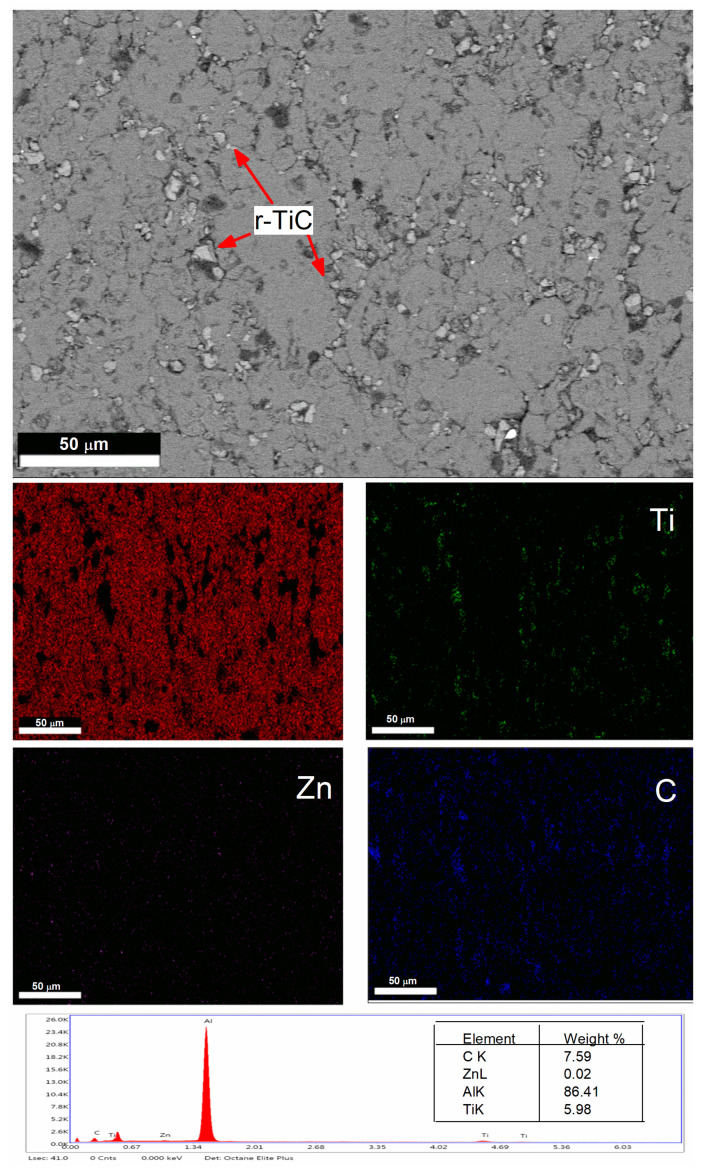
FESEM and EDS-elemental mapping of the AlZnMgCu + 14% r-TiC composite.

The microstructure of the nine composites is shown in Figure 4a–i. In Figure 4a, the microstructure of the 0% r-TiC showed low porosity compared to the other composites visually. The 2–8% r-TiC reinforced composites revealed a uniform distribution of reinforcement on the grain boundaries of the AlZnMgCu matrix. With 10% r-TiC addition, low agglomeration started and increased with the increase in r-TiC addition. Figure 4i shows the heavy agglomeration of r-TiC during 16% addition. The good bonding between the matrix and the reinforcements improved the composites’ strength, but the presence of these agglomerations affected the bonding strength and frequently diminished the mechanical characteristics of the composites. According to the findings of Manohar et al. [29] and Bodukuri et al. [34], clusters or agglomerations were formed due to variations in the concentrations of the matrices and ceramic particles. Also, the uniform distribution of r-TiC and their presence was confirmed through the FESEM with EDS mapping of the AlZnMgCu +14% r-TiC composite, as shown in Figure 5.

#### 3.2.2. Density and Porosity

**Figure 6:**
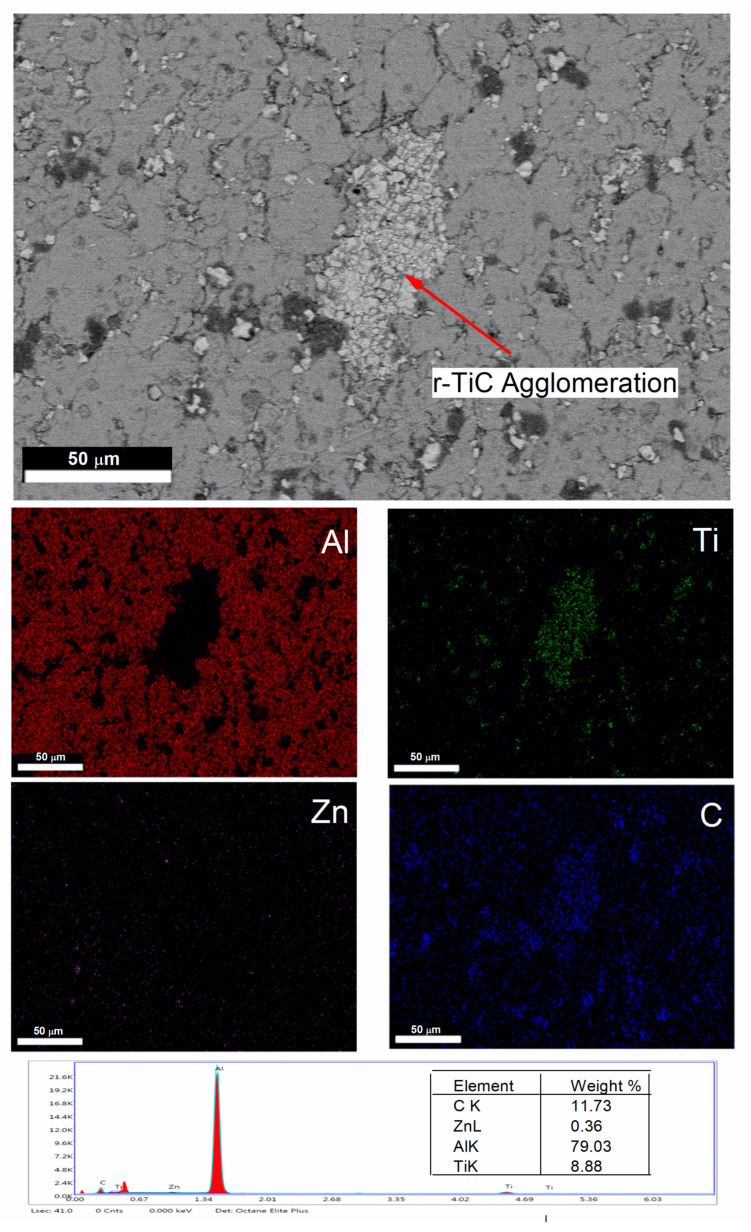
FESEM and EDS-elemental mapping of the AlZnMgCu + 16% r-TiC composite.

When the porosity is less than 5%, it will facilitate the improvement of the compressive strength and hardness; when obtaining beyond 5% porosity, the foreign particle inside the composite will come into play and initiate early crack propagation, which in turn reduces the compressive strength [35]. In the current research, the r-TiC reinforcement was added with steps of 2 weight percent (wt. %) from 0 to 16 weight percent. For each reinforcement percent addition, i.e., 0, 2, 4, 6, 8, 10, 12, 14, and 16, the porosity was measured in % and observed as 3.0, 3.5, 3.9, 4.0, 4.1, 4.5, 4.8, 5.0, and 5.2, respectively. Density increased with the increase in r-TiC addition, reaching its maximum for 2.774 g/cm^3^ for 16% r-TiC due to the density difference between the matrix (theoretical density—2.81 g/cm^3^) and the reinforcement (theoretical density—4.93 g/cm^3^). The sintered composite showed a low porosity of 3% for a 0% r-TiC addition and a high porosity of 5.2% for a 16% r-TiC addition. Due to variations in their deformation capabilities, the matrices and reinforcements worked together to create pores. The hard ceramic particles comprising the agglomerations and clusters created spaces that served as the pre-existing fissures, as shown in Figure 6, in the FESEM with EDS mapping of the 16% r-TiC added composite. The thermal imbalance between the matrix and the reinforcement particles was another critical component in pore development [36]. Hence, pores were formed at the interfaces during sintering due to differing thermal expansion rates.

#### 3.2.3. Hardness and Compressive Strength

Notably, the blending and sintering processes were crucial to achieving grain boundary and dislocation-strengthening mechanisms in the composite materials, contributing substantially to their overall strength [20]. When a hard second phase was introduced into a softer matrix, according to Orowan’s theory, it functioned as a barrier that restricted the movement of dislocations. These secondary phases encountered dislocations, which became immobilized in their path. For dislocations to pass through precipitates or reinforce phase particles, which are typically composed of ceramics or intermetallics, significant forces are required [21]. In either circumstance, these obstacles inhibit dislocation movement, increasing hardness. Moreover, when high stress values are applied, the dislocations effectively pass through the obstacles. They then form new dislocation loops around the particles, further impeding the motion of subsequent dislocations, which ultimately leads to a substantial increase in the material’s hardness and compressive strength. Also, small grain sizes formed during the process promote Orowan strengthening.

**Figure 7:**
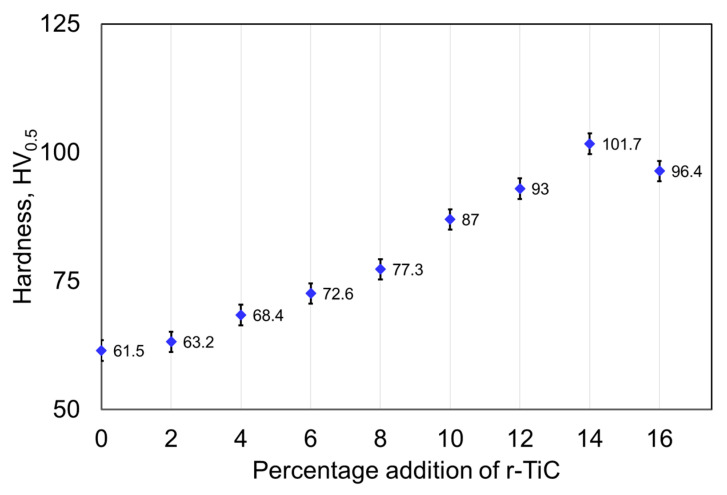
The hardness of AlZnMgCu + Y% r-TiC composites.

Vicker’s hardness with the standard deviation measured for the sintered composites, as illustrated in Figure 7, displays the significance of the r-TiC addition to the AlZnMgCu matrix. A remarkable hardness improvement in the AlZnMgCu matrix was seen with the addition of r-TiC particles. It was evident that all composites had a higher hardness than the 0% r-TiC addition. The 14% r-TiC exhibited the highest hardness of 101.7 HV0.5, increasing by 66% over the 0% r-TiC samples. But the 16% r-TiC addition had a 9% hardness, less than that of the 14% r-TiC addition, and the hardness increased by 6% as compared to the hardness of the 12% r-TiC addition. When TiC particles were added to a matrix material in high percentages, particle agglomeration was risky and could lead to reduced hardness and other adverse effects.

**Figure 8:**
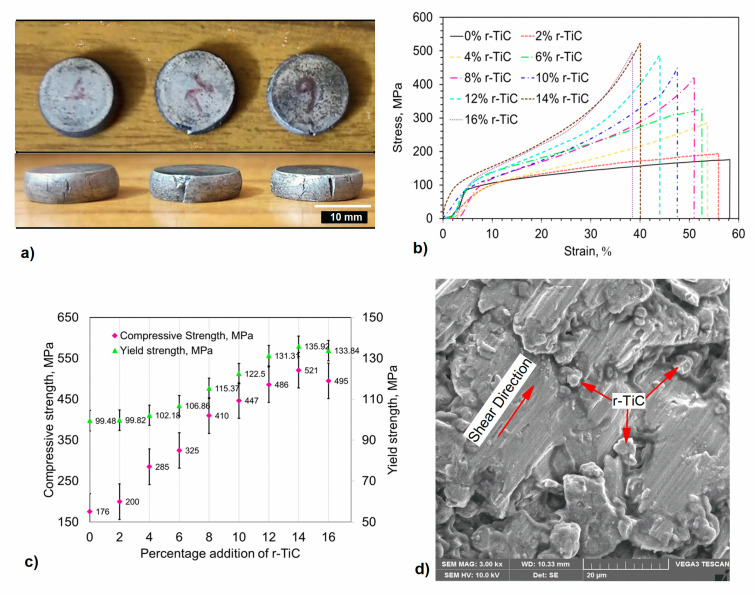
(**a**) Compression-tested samples AlZnMgCu + 14% r-TiC. (**b**) Stress vs. strain curve for compression test of AlZnMgCu + Y% r-TiC composites. (**c**) Compressive and yield strength of AlZnMgCu + Y% r-TiC composites. (**d**) SEM image of AlZnMgCu + 14% r-TiC composites with compressive fractured surfaces.

Figure 8a depicts the compression test results and crack initiation using the AlZnMgCu + 14% r-TiC composites with the ultimate compression strength. Figure 8b gives the stress vs. strain percentage curve for the AlZnMgCu + Y% r-TiC composites during the compression test at a 1 mm/min strain rate. Figure 8c reveals the compression and yield strength with the standard deviation of the composites developed by adding recycled reinforcement (r-TiC). The progressive incorporation of r-TiC reinforcement into the AlZnMgCu matrix gave rise to a 69% spike in the compressive strength in the AlZnMgCu +14% r-TiC composite compared to the 0% r-TiC addition. Compression strength was improved by the enhanced molecular-level bonding of the matrix and the reinforcement particles, as well as the homogenous distribution of the reinforcement over the matrix [37]. Porosity was found to be directly related to compressive strength. Based on Saravanan et al. [35], it is understood that, as the porosity is high, the compressive strength is also impressive up to the threshold point of reinforcement addition. In our study, we understood that 14% reinforcement r-TiC showed better compressive strength than 16% r-TiC. The better compression strength was due to increased plastic deformation and grain boundary strengthening. Many studies have shown that compressive strength can be increased by adding reinforcement content [38]. Increased compressive strength can be credited to the uniform and fine incorporation of r-TiC particles into the matrix and around the grain boundaries. Figure 8d exhibits the homogeneous distribution of the r-TiC in the AlZnMgCu matrix of a 14% r-TiC added sample, which exhibited the highest compressive strength of 521 MPa. When loads were applied, a strong interface connection prevented plastic deformation, which further enhanced the hardness of the composite and facilitated effective load transmission mechanisms between the matrix and the reinforcement particles. Further, shearing happened, as shown in Figure 8d, in order for the AlZnMgCu +14% r-TiC composite to establish the Orowan strengthening mechanism and to ensure the highest compressive strength. It was also essential to subject green compacts to high temperatures throughout the sintering process to ensure that the diffusion and bonding processes were carried out correctly [39]. Due to this, the high-temperature stimulation energies of the particles increased, causing the nearby matrix and reinforcements to react chemically, leading to the formation of intermetallic compounds like MgZn_2_, which thus improved mechanical properties.

After a 14% r-TiC addition, an increase in the agglomeration of the reinforced particles resulted in a loss in the composite’s mechanical characteristics [36]. Additionally, based on similar results, it could have been caused by inadequate binding due to a lack of wetting at the interface of the particles and the base metal, as well as a failure of focused load transmission due to the random arrangement of the reinforcement particles [40].

#### 3.2.4. Wear Analysis

**Figure 9:**
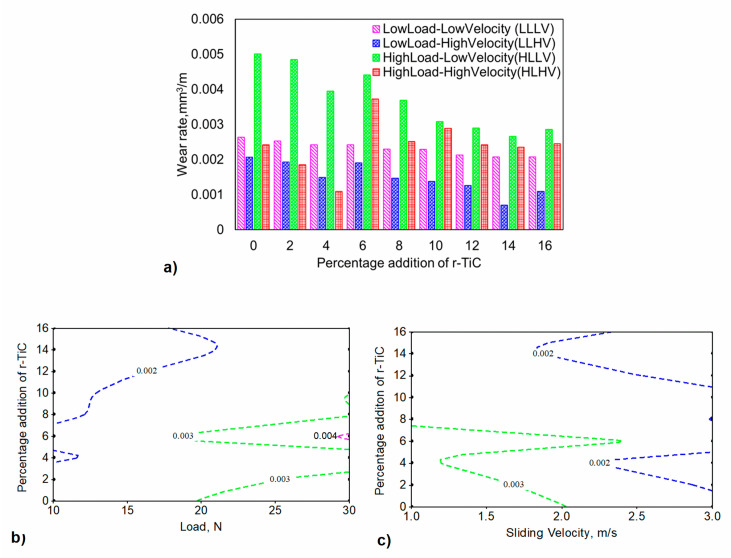
(**a**) Wear rate of AlZnMgCu + Y% r-TiC composites. (**b**) Contour plot for wear rate of AlZnMgCu + Y% r-TiC composites considering the load and percentage addition of r-TiC. (**c**) Contour plot for wear rate of AlZnMgCu + Y% r-TiC composites considering the sliding velocity and percentage addition of r-TiC.

The wear rate for the developed composites was evaluated by the mass loss method. Each composite was subjected to a wear test in all extreme combinations of LLLV, LLHV, HLLV, and HLHV. As a result, Figure 9a depicts the wear rate for all nine composites when subjected to four different wear test parameter combinations. The results show that the 0% r-TiC-added composite at HLLV exhibited the highest wear rate (0.00501 mm^3^/m), while the 14% r-TiC-added composite at LLHV exhibited the lowest wear rate (0.0007 mm^3^/m). The wear rate decreased with an increase in the percentage of r-TiC up to 14% in r-TiC, but increased in the 16% r-TiC-added composite. However, there was a wear rate dip in the 4% r-TiC addition at HLHV, possibly due to the three-body abrasion between the pin, disk, and debris [41]. The 6% r-TiC-added composites showed a spike in wear rate in all wear conditions, while the wear rate fell for the 8% r-TiC-added composites. Due to the insignificance of r-TiC addition at LLLV, r-TiC addition above 8% resulted in a similar wear rate [42]. The mechanisms behind such variations will be explained further. The contour plots in Figure 9b,c illustrate the impact of load and sliding velocity on wear rate considering the r-TiC percentage. The wear rate increased with a rise in load for a low percentage of r-TiC addition. For higher addition percentages, even at higher loads, they bore the load and exhibited reduced wear rates of less than 0.003 mm3/m. From 8% to 16%, r-TiC addition exhibited a lower wear rate of less than 0.002 mm3/m up to a 20 N load. The wear rate decreased with a rise in the sliding velocity for all r-TiC-added composites. The lower sliding velocity led to a higher wear rate of up to 8% in r-TiC. At slower sliding speeds, more time was spent in contact with the rough edges of the surfaces. This increased the number of microwelds, which required more energy in the sliding region to wear off, causing mass loss. The obtained wear rate for AlZnMgCu + 14% r-TiC composites was compared to the results of the previous study conducted by Sarvanan et al. [16], and it was found that AlZnMgCu + 14% r-TiC showed better wear resistance than the AA7075 + 16% TiC composite’s wear rate when operated under the same wear study conditions of a 9.81N load and a 3 m/s sliding velocity. The AlZnMgCu + 14% r-TiC outperformed it by 20% due to the varying particle size of the in situ-developed r-TiC and the advantage of the powder metallurgy process, which allowed for homogenous distribution of r-TiC in the AlZnMgCu matrix during blending.

#### 3.2.5. Friction Analysis

**Figure 10:**
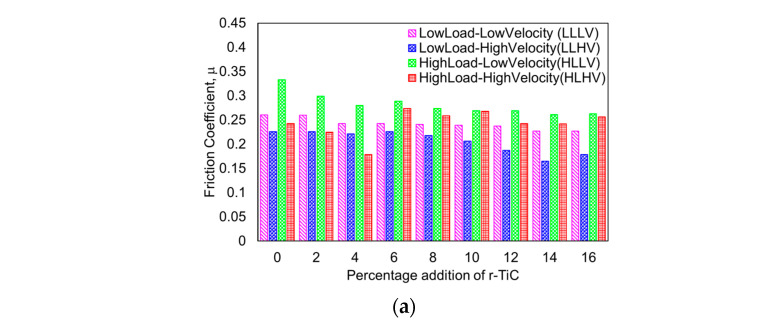

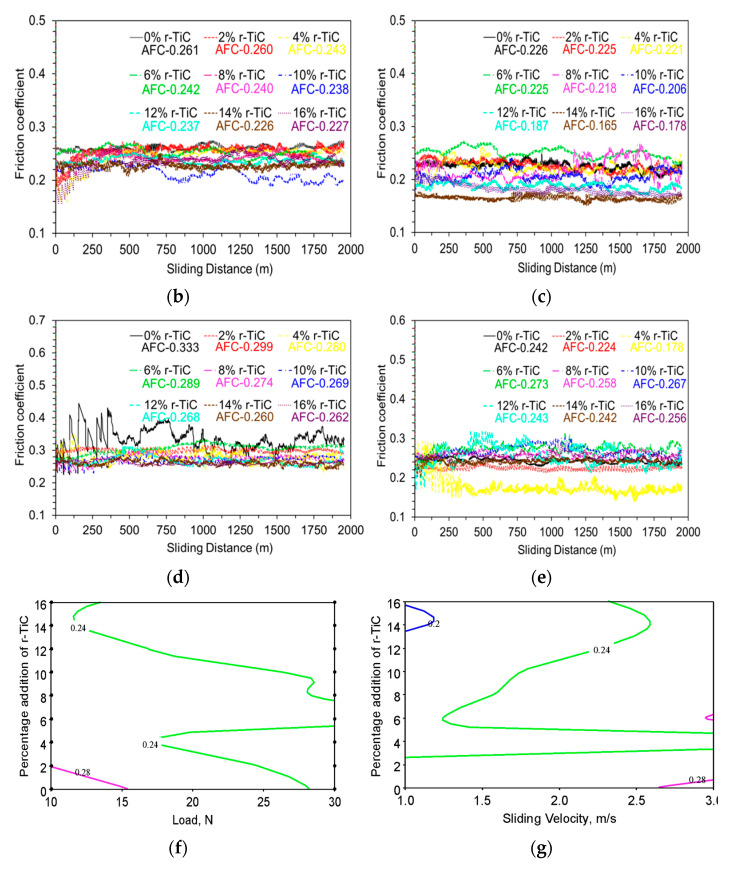
(**a**) Friction coefficient of AlZnMgCu + Y% r-TiC composites. (**b**) Contour plot for friction coefficient of AlZnMgCu + Y% r-TiC composites considering the load and percentage addition of r-TiC. (**c**) Contour plot for friction Coefficient of AlZnMgCu + Y% r-TiC composites considering the sliding velocity and percentage addition of r-TiC. Friction coefficient curves of AlZnMgCu + Y% r-TiC composites: (**d**) under low-load and low-velocity (LLLV) conditions; (**e**) under low-load and high-velocity (LLHV) conditions; (**f**) under high-load and low-velocity (HLLV) conditions; and (**g**) under high-load and high-velocity (HLHV) conditions.

In metal–matrix composites, friction was affected by reinforcement, the average interparticle gap, interfacial bonding with both the matrix and the particle, surface hardness, contact area, and the particle’s resistance to shear dislocation. The friction coefficient (FC) was calculated using the frictional force data collected and the load applied during the wear test, as shown in the bar chart (Figure 10a). The friction coefficient, also known as the average friction coefficient (AFC), represents the average friction force during the 2000 m sliding distance used in the calculation. For the LLLV wear condition, the friction coefficient, as shown in Figure 10b, decreased with a rise in r-TiC addition due to its lubrication functionality. As shown in Figure 10c, the friction coefficient in the LLHV wear condition remained constant up to 6% r-TiC addition, then decreased until 14% r-TiC addition and again increased for the 16% r-TiC-added composite. Under the HLLV wear condition, the friction coefficient in Figure 10d showed a similar decreasing trend with increasing r-TiC addition, but peaked at the highest point among all wear conditions, resulting in poor wear resistance [43]. Finally, at HLHV, as shown in Figure 10e, the coefficient did not reveal the same trend as in the other conditions. It initially showed a decrease and spiked at a 6% r-TiC addition. It further exhibited a down-and-up trend for the remaining r-TiC percentage additions. The contour plots in Figure 10f,g explain the impact of load and sliding velocity on the average friction coefficient in relation to the r-TiC percentage, respectively. Up to 2% r-TiC, the 15 N load initially showed a high friction coefficient greater than 0.28. For all percentage additions at low loads, the coefficient of friction was around 0.24. A higher percentage, i.e., above 8% r-TiC addition in all loading conditions, revealed a lower coefficient of friction of around 0.22. When considering sliding velocity, initially, at a high velocity and low percentage, it exhibited a high friction coefficient. For all percentages of sliding speed, the average friction coefficient was between 0.22 and 0.26.

#### 3.2.6. Wear Mechanisms

**Figure 11:**
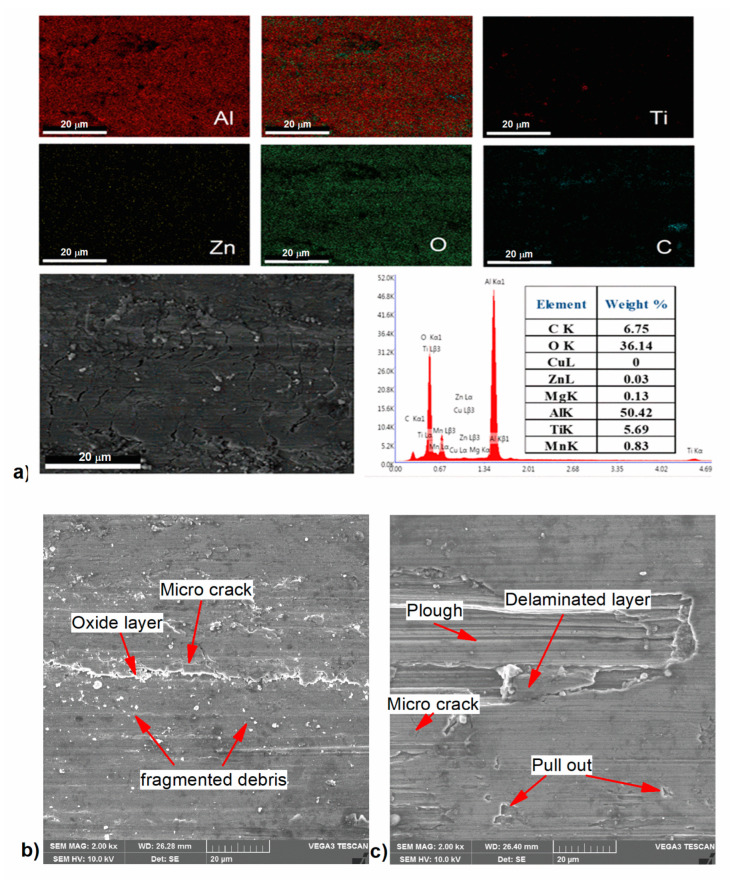
(**a**). Elemental mapping of the worn surface of AlZnMgCu + Y% r-TiC showing oxide layer; (**b**) SEM image of low-worn AlZnMgCu + 14% r-TiC composite surface; (**c**) SEM image of high-worn AlZnMgCu + 6% r-TiC composite surface.

Wear mechanisms like delamination, surface grooves, micro-cracks, and plows were all features of the worn surface. The presence of an oxide layer was observed on the wear surface of the AlZnMgCu + 14% r-TiC composite, which was confirmed by elemental mapping analysis using SEM with EDS, as shown in Figure 11a. Also, the absence of deep grooves on the wear surface was evidence for improving the specimen’s wear resistance. The higher hardness of the 14% r-TiC composite helped in increasing the wear resistance. Also, the formation of an oxide layer confirmed surface oxidation, and the presence of an oxidative wear process supported the improvement of the wear resistance. Fractures in the matrix near the r-TiC particles were initiated due to sliding at a high load, and when the cracks were propagated parallel to the sliding direction, the matrix broke up and debris accumulated. Figure 11b demonstrates how the oxide layer was formed to protect the underlying surface from wear and how the harder surface of the specimen reduced the abrasive action of the detached oxide particles, resulting in exceptional wear resistance. Figure 11c shows the AlZnMgCu + 6% r-TiC composite, which was subjected to the delamination caused by microcracks, fracturing the surface layers due to severe plastic deformation with the maximum wear rate. This adhesive wore away a plumb of material which, in turn, also increased the wear rate for the 6% r-TiC samples [44]. It was clear from the discussion that the 14% r-TiC-added composite outperformed all other developed composites based on the mechanical and tribological properties.

#### 3.2.7. Corrosion Analysis

**Figure 12:**
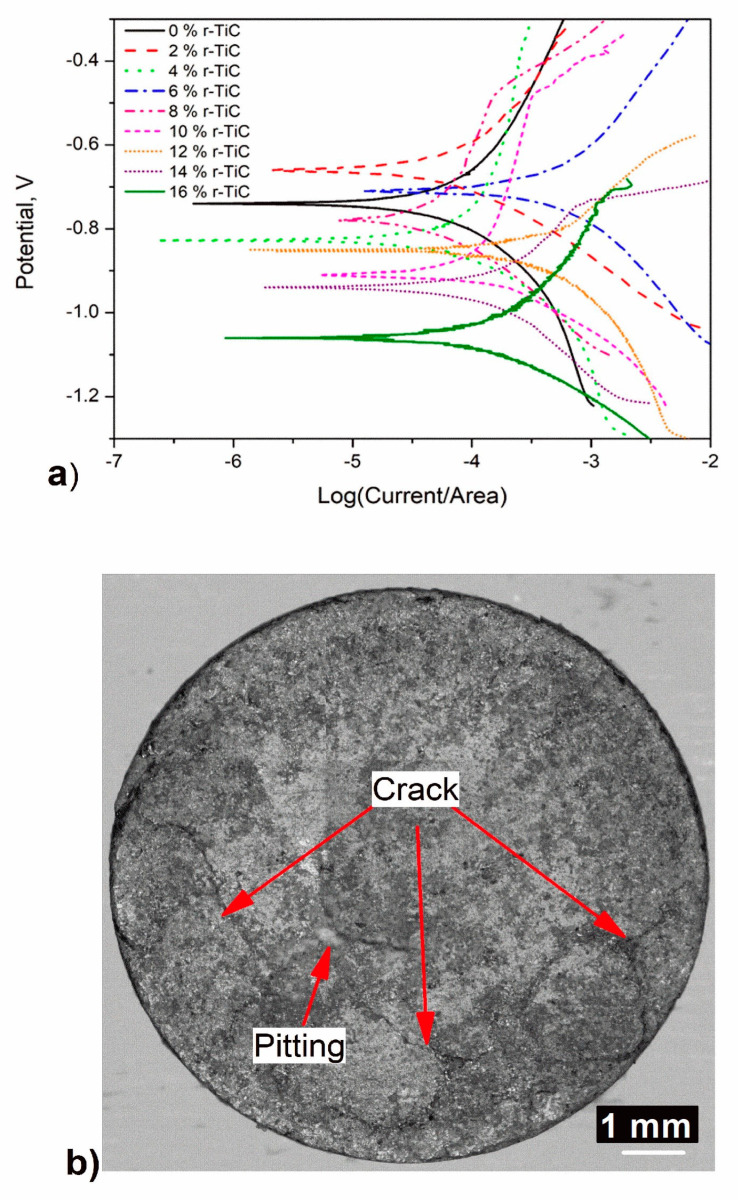
(**a**) Tafel plot of AlZnMgCu + Y% rTiC composites. (**b**) Macroscopic image of the corroded surface of AlZnMgCu + 14% r-TiC composite.

The corrosion resistance study of AlZnMgCu +Y% r-TiC composites was carried out to establish the corrosion behavior with r-TiC addition. Figure 12a depicts the Tafel plot obtained from the PDP corrosion test, the corrosion potential (Ecorr), and the current density (Icorr) extracted from the plot, as tabulated in Table 1. The corrosion rate in miles per year was calculated using the obtained current density (Icorr), which is tabulated in Table 1. The results reveal that the corrosion rate improved for the 2% r-TiC compared to the 0% r-TiC by 22.2%, and that further addition greatly reduced the corrosion rate from 0.5108 to 57.028 mpy for the 16% r-TiC added composite. A similar result was seen when Al2O3 was added beyond 1.5% to the AA7075 matrix [45].

There were various reasons why the corrosion resistance of the AlZnMgCu base alloy decreased when the rTiC concentration increased. One theory suggests that the inclusion of r-TiC particles, despite r-TiC’s reputation as a corrosion-resistant material and insulator, enhanced the corrosion rates as they disrupted the continuity of the AlZnMgCu matrix. The result was the formation of corrosion-attack hotspots. Several reports on the corrosion behavior of Al-matrix composites have noted this trend [46]. Researchers also examined the corrosion behavior of aluminum composites with reinforcement particles and found that the presence of reinforcement particles in the metal matrix did not increase corrosion, but that the size, homogeneity of reinforcement distribution, microstructure, and interfacial response among the matrix and the reinforcement all played essential roles [47]. Additionally, it was noticed that the oxide layers on the aluminum metal surface were affected when ceramic reinforcement was added to the base alloy. As they became increasingly disjointed in the initial layers of the damaged surface, the surface’s resistance to further corrosion was reduced. Therefore, the presence of cathodic impurities, such as intermetallic or reinforcing particles, accelerated corrosion. It was generally recognized that the vicinity of r-TiC was more susceptible to corrosion due to its cathodic nature. A macro image of the corroded surface of the AlZnMgCu + 14% r-TiC composite is shown in Figure 12b, revealing pitting and crack initiation on the surface due to agglomeration of r-TiC when higher percentages of reinforcement were added. It was further understood that adding r-TiC beyond 2% was critical, so corrosion studies could be performed for low percentage additions of r-TiC, for example, in steps of 0.25% up to 2.5%. Low percentage addition will reveal a new perspective on the addition of r-TiC with respect to corrosion.

## 4. Conclusions

The r-TiC was successfully used as a reinforcement to fabricate the AlZnMgCu metal matrix composite and to study the mechanical, tribological, and corrosion properties. The results show that the r-TiC was successfully incorporated into the AlZnMgCu matrix, and the r-TiC composites achieved better mechanical and tribological properties than those of the regular available TiC composites. MMCs with 14% r-TiC addition outperformed all other percentage additions in mechanical and tribological testing by 60%. However, the corrosion study revealed that the addition of 2% r-TiC corrosion behavior showed 22% superior corrosion resistance to other percentage additions. Thus, based on application and property requirements, the percentage of r-TiC can be used. This article provides clear confidence that a TiC produced with a low-cost route (in situ stir casting) can be recovered and reused as a reinforcement. This can be performed equivalently or even better in certain properties during addition to the AlZnMgCu matrix.

### Future Scope

The results of the current research provide the basic results regarding the selected materials—recycled titanium carbide and AlZnMgCu—for the selection of a material for suitable application as per industrial needs. Hence, the authors are planning to conduct application-based research in the future for the AlZnMgCu + Y% r-TiC composite developed in our lab. Further in the future, the 14% r-TiC added composite can be subjected to secondary processes like forging, extrusion, etc., to improve the density. Also, the synthesized composite can be subjected to heat treatment to improve its properties as a sintered and secondary processed composite to gain a better understanding of its characteristics.

## Figures and Tables

**Table 1:** Corrosion results of theAlZnMgCu + Y% r-TiC composites.

S. No.	Percentage Reinforcement of r-TiC (wt. %)	Potential, Ecorr (V vs. SCE)	Current Density, Icorr (µA/cm^2^)	Corrosion Rate, mpy
1	0	−0.740 ± 0.02	1.25 ± 0.10	0.6119 ± 0.02
2	2	−0.660 ± 0.01	1.02 ± 0.09	0.5108 ± 0.01
3	4	−0.870 ± 0.03	4.25 ± 0.18	2.0330 ± 0.09
4	6	−0.710 ± 0.02	3.98 ± 0.15	2.0386 ± 0.09
5	8	−0.780 ± 0.02	12.5 ± 0.25	6.5469 ± 0.12
6	10	−0.910 ± 0.03	29.9 ± 0.42	13.9730 ± 0.19
7	12	−0.850 ± 0.03	39.0 ± 0.45	20.8811 ± 0.24
8	14	−0.940 ± 0.04	67.3 ± 0.52	36.8260 ± 0.45
9	16	−1.060 ± 0.04	102.0 ± 1.25	57.0276 ± 0.54

## Data Availability

The original contributions presented in the study are included in the article, further inquiries can be directed to the corresponding authors.
